# Thermodynamic considerations in renal separation processes

**DOI:** 10.1186/s12976-017-0048-7

**Published:** 2017-01-26

**Authors:** Robert H. Louw, David M. Rubin, David Glasser, Robyn F. R. Letts, Diane Hildebrandt

**Affiliations:** 1 Materials and Process Synthesis (MaPS) Research Unit, University of South Africa, Pioneer Avenue, Roodepoort, 1709 South Africa; 2 Biomedical Engineering Research Group, School of Electrical and Information Engineering, University of the Witwatersrand, 1 Jan Smuts Avenue, Johannesburg, 2000 South Africa

**Keywords:** Human kidney, Urine production, Separation work, Gibbs energy, Energy balance, Sensitivity analysis

## Abstract

**Background:**

Urine production in the kidney is generally thought to be an energy-intensive process requiring large amounts of metabolic activity to power active transport mechanisms. This study uses a thermodynamic analysis to evaluate the minimum work requirements for urine production in the human kidney and provide a new perspective on the energy costs of urine production. In this study, black-box models are used to compare the Gibbs energy inflow and outflow of the overall kidney and physiologically-based subsections in the kidney, to calculate the work of separation for urine production.

**Results:**

The results describe the work done during urine production broadly and for specific scenarios. Firstly, it shows glomerular filtration in both kidneys requires work to be done at a rate of 5 mW under typical conditions in the kidney. Thereafter, less than 54 mW is sufficient to concentrate the filtrate into urine, even in the extreme cases considered. We have also related separation work in the kidney with the excretion rates of individual substances, including sodium, potassium, urea and water. Lastly, the thermodynamic calculations indicate that plasma dilution significantly reduces the energy cost of separating urine from blood.

**Conclusions:**

A comparison of these thermodynamic results with physiological reference points, elucidates how various factors affect the energy cost of the process. Surprisingly little energy is required to produce human urine, seeing that double the amount of work can theoretically be done with all the energy provided through pressure drop of blood flow through the kidneys, while the metabolic energy consumption of the kidneys could possibly drive almost one hundred times more separation work. Nonetheless, the model’s outputs, which are summarised graphically, show the separation work’s nuances, which can be further analysed in the context of more empirical evidence.

## Background

Human kidneys consume a large amount of oxygen representing substantial metabolic energy: approximately 21.3 W/kg, which is equal to that of the heart and more than the brain [36]. In addition to the energy from metabolic processes, the kidneys receive energy in the form of pressurised blood, by taking 20% of the cardiac output [34]. While the energy consumption of the organs has been established and while multiple mechanisms have been studied [15], what has not been established clearly is the minimum work required for the kidneys to produce urine.

This paper is aimed at providing an alternative perspective to urine production, as opposed to studying specific mechanisms and metabolic energy consumption. It focuses on the work done in the broader urine production processes and outlines the relationship between excretion rates and minimum work requirements, which may be characteristic of the system.

A number of studies have investigated the work required of the human kidneys. Newburgh [22] built on a study from 1905 by Ladislaus van Rhorer, which aimed to determine the osmotic work of the kidneys for different amounts of water and urea excretion. Newburgh’s study was nonetheless limited, as additional analysis could have provided a more comprehensive understanding of the relationship between water and urea excretion, and could also have considered the effect of other solutes. Later, Weinstein [38] created a more advanced model using the concept of Gibbs energy (G) and chose to focus his study on a rudimentary representation of the counter-current multiplication system of the kidney within the renal medulla. Other studies [33, 35], similarly limited to the medulla, have investigated renal energy requirements, albeit with more sophisticated models. This paper builds on this research to determine the energy requirements for urine production by the overall system under a wide variety of conditions, without considering specific mechanisms.

### Background to the operation of the kidney

The model used in this study is based only on the specifications of the inlet and outlet streams and we consider only the work required to achieve the separation of the urine from the blood. At such a high-level, the particular mechanism of separation is not important, but it is possible to determine the lowest possible work cost of separating urine from blood. We will thus use an abstract representation of the renal system. Nonetheless, we will mention some of the current uncertainties in the field, such that they may be taken into account when evaluating the results.

The kidney is a separation system designed to maintain homeostasis: blood circulating through the body is kept at near constant solute concentrations, through the removal of waste and excess substances by the kidney [29]. On average, one pair of human kidneys processes approximately 900 litres of blood plasma to produce 2 litres of urine per day. In the process the liquid, which starts with a concentration of 290 mOsm/l, is concentrated up to 1200 mOsm/l [9]. Urea is the primary waste product excreted by mammalian kidneys and its concentration in urine is two orders of magnitude higher than in blood. (More data are presented and referenced in the section titled Input data). Urea excretion counterbalances the nitrogen released during the breakdown of proteins, such that the body can maintain a nitrogen balance. Other substances, such as water, sodium and potassium, need to be excreted when they are in excess and retained when they are below optimal concentration [9].

In their review of the nephrology literature, Sands and Layton [29] show that the collective understanding of the mechanism of renal urine production remains incomplete, particularly in the inner medulla. The kidney is generally thought to produce urine through a system of active and passive mass transfer mechanisms, where the active mechanisms utilize metabolic energy to drive mass transfer and the passive mechanisms utilize mass transfer driven by convection or diffusion [29]. With both types of mechanisms, there are uncertainties. For example, Layton and Layton [14] have criticised the counter-current multiplication paradigm, first proposed by Kuhn and Ryffel [13]. The objection is that a new model is required to take into account recent discoveries pertaining to the renal membrane structures and to mitigate inaccuracies in how the cortico-medullary concentration gradient is created. Other researchers are also investigating mechanisms by which the kidney can apply metabolic energy, for example, through physical contractions of the pelvic wall [31] or through inner medullary lactate production [10].

## Methods

The methodology described below is similar to practises commonly used in the study of industrial chemical processes. Our approach uses limited renal mass flow and concentration data from the literature to determine the mass flow rates and concentrations of all the streams of the model, through mass balance calculations. Once the mass flow has been calculated, one can then determine the minimum work required for separating urine from blood, using thermodynamic principles to quantify the Gibbs energy (G) associated with the streams.

**Fig. 1:**
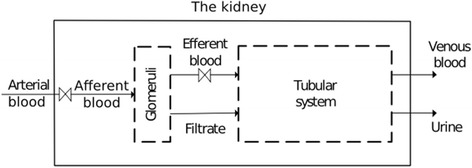
The black-box models of the kidney used in this study, showing the overall black-box model (solid borderline), which is then divided into two mass-transfer processes (dashed borderlines): filtration in the glomeruli and reabsorption in the tubular system. The valve symbol (▻◅) indicates positions where there is a pressure drop due to friction in the kidney

We have modelled the kidneys on two levels of abstraction: one where the two kidneys are represented by a single black box; the other where the urine production process is split into two parts, each represented by a different black box (see Fig. 1 below). These black-box models only consider the mass and energy flows into and out of the processes. By employing this approach, we can understand the overall thermodynamic constraints on the system without needing to understand the details of the sub-processes inside the system. The calculation determines the target, or minimum work requirement, for the kidneys to produce urine. However, it must be noted that the internal structures and functions in the kidney are expected to introduce inefficiencies, which would increase the work requirement of the process.

The model represents kidneys operating at steady state, which entails assuming there is no depletion or accumulation of mass or energy. By making this assumption we can use daily volumetric flow data for the calculations.

The first level of the model, depicted as the solid borderline in Fig. 1, pertains to the overall kidney. It includes the arterial blood flowing into the kidney and the venous blood and urine flowing out. In this overall model, the two kidneys are grouped together as a single process, which is feasible given that they operate in parallel. With this configuration of the model, the relatively small lymph stream [19] is combined with the outflow of venous blood. More research outside the scope of this study is required to collect data relating lymph concentrations and urine concentration.

The second level of the model, outlined with the dashed borderlines in Fig. 1, shows how the overall process is split into two processes in series. The first step of urine production is filtration by the glomeruli, as indicated in the first sub-process in Fig. 1. In the glomeruli, blood is split into protein-rich efferent blood, and protein-free glomerular filtrate. The second sub-process is reabsorption. Here the glomerular filtrate is partially reabsorbed to leave behind concentrated urine. Any secretion that occurs in the second sub-process is included in the net mass transfer, since the black-box model chosen for this study only considers the total difference between inflowing and outflowing streams. Moreover, substances secreted into the filtrate (for example, hydrogen ions, organic acids and bases) have not been included and the energy associated with these substances is assumed to be negligible, regardless of their increase in concentration levels, because of their low concentrations in the inlet and outlet streams (see [20]). We will refer to the structure performing this second function, the recovery of filtrate, as the “tubular system.” It includes the kidney’s renal tubules, the renal interstitium, and capillaries.

In summary, the first level of the model contains no information regarding the internal processes in the kidney, while only the most basic process steps are included in the second level of the model. In addition, the valve symbols in Fig. 1 indicate regions in the kidney that are known to have large pressure drops due to friction. The pressure energy lost to friction is not available for separation work and its inclusion in the model will be discussed.

### Input data

There are sufficient published data describing flow rates and concentrations in the human renal system to perform the theoretical calculations. This data, together with the assumptions made, will be discussed next. The reader may consult the Appendix for a summarised list of assumptions.

#### Concentration data

The main components of blood plasma and their concentrations are listed in Table 1. Although red blood cells and other similarly large structures constitute a significant fraction of the total volume or mass of blood, these components are so large that they do not have significant molar concentrations. Consequently, they do not contribute significantly to the mixing or entropy in the mixture, and therefore, are assumed negligible in the calculations.

**Table 1:** Collected blood plasma concentration data [6, 9, 20, 25, 26]

Substance	Reference range (mmol/l)	Mean blood plasma concentration (mmol/l)
Sodium (Na ^+^ )	136-145	142
Potassium (K ^+^ )	3.5-5	4.2
Calcium (Ca^2 +^ )	1.3-2.6	1.4
Chloride (Cl ^−^ )	98-108	108
Bicarbonate (HCO_3_^−^ )	23-28	25.5
Glucose	3.9-5.8	5.6
Urea	2.9-7.1	4
Creatinine	0.062-0.115	0.088
Proteins	0.718	0.718
Total	269.4-302.3	291.5

The solute excretion rates used in the model are shown in Table 2. This data can be used to calculate urine concentrations, given the 24-h urine production volume. When interpreting the results of the model, it is important to remember the maximum concentration of human urine expected from empirical studies is 1.2 Osm/kg water [29].

**Table 2:** Urine solute excretion rates used to calculate urine concentrations [2, 9, 21]

Substance	Reference range (mmol/day)	Excretion rate used (mmol/day)
Sodium (Na ^+^ )	100 – 260	180
Potassium (K ^+^ )	25 – 100	63
Calcium (Ca^2 +^ )	2.5-7.5	5
Chloride (Cl ^−^ )	80–250	165
Bicarbonate (HCO_3_^−^ )	9.9–16.6	13
Glucose	0	0
Urea	200–405	303
Creatinine	2	2
Total	419–1023	731

The issue of electro-neutrality deserves some attention. The authors decided to use mean solute concentrations for urine, rather than an arbitrary combination of other concentrations that would be closer to electrolytic neutrality. Adjusting the rates of sodium and chloride excretion in the model (using the link in at the end of the paper) to achieve electro-neutrality could affect the results by as little as 1 mW. There are, however, other factors to consider. On the one hand, the concentrations that give electro-neutrality cannot be determined precisely without a significant increase in the complexity of the model, to take into account the charge of proteins. On the other hand, when adjusting the excretion rates relative to the established baseline, it is easy to ensure that for every mole of monovalent positive ions transferred, one mole of monovalent negative ions must also be transferred, such that the electric charge of the solution does not increase. Any changes made to the amount of sodium or potassium ions excreted when doing the sensitivity analysis is therefore accompanied by equal changes in the amount of negative ions excreted.

The concentration of water was also considered in the model. The molar concentration of pure water at a temperature of 37 °C is 55.14 mol/l [27] and this changes marginally when the water contains small solutes. For protein-free solutions, like the filtrate, we assumed that smaller solutes, such as urea, occupy no space. We therefore approximated the concentration of water in the filtrate and urine streams to be equal to that of pure water at 37 °C. For blood plasma, however, the space occupied by plasma proteins is substantial: the water concentration in blood plasma is calculated as 52.7 mol/l, given the protein data from Table 4 in the Appendix.

#### Pressure data

The pressure in the kidney varies throughout the day: the kidney controls the pressure drop through vasoconstriction at various points and thereby manipulates flow rates [9]. For the purposes of this study, the pressure drops are assumed constant, as in Table 3.

**Table 3:** Human pressure data used [4, 9, 23, 34]

Point	Pressure (mm Hg)
Arterial blood	100
Afferent blood	60
Efferent blood (before pressure losses)	59
Efferent blood (after pressure losses)	42
Filtrate	18
Venous blood	8
Urine	8

As blood flows through the kidney, its hydrostatic pressure drops, which can either be used to overcome friction as the fluid flows along a tube or for separation work, such as filtration. It is unclear what fraction of the energy associated with pressure drop is used to drive flow and what is used to concentrate urine. Pressure measurements have been reported at various points in the kidney in the literature and when looking at the structure of the kidney, some of these measured pressure drops must be mainly due to friction losses as there is thought to be no separation occurring over these sections of the kidney. For this reason we assumed in the model that the energy associated with these pressure losses cannot perform work for filtration or for the recovery of filtrate. These losses are marked by the valve symbol in Fig. 1. In the model, the pressure drop due to stream splits and friction before the glomeruli is taken as 40 mm Hg [9, 23]. Inside the glomeruli, the pressure drop on the blood side (in the glomerular capillaries) is taken as 1 mm Hg [9, 38], and the hydrostatic pressure gradient between the blood and filtrate in the glomeruli is set at 32 mm Hg [9]. Pressure losses along the efferent arteriole, before the peritubular capillaries and the vasa recta are approximated as 17 mm Hg [23]. Further data are provided in Table 3.

#### Filtration data

The last input component required by the model is the filtration fraction of the glomerulus. The available data show that approximately 900 l/day of plasma is processed to produce 180 l/day of filtrate [23, 34]. This translates into a filtration fraction of 20% and we kept this fraction constant in all our calculations. We further assumed that all solute concentrations in the filtrate and the efferent blood are the same, immediately after the glomeruli, while no plasma proteins pass into the filtrate [9].

### Calculations

The model used in this study, with calculation steps described below, can be downloaded using the link given in at the end of the paper. Also, for the reader’s convenience, all of the assumptions made while using the equations below are listed in the Appendix.

#### Mass flow calculations

The above data are sufficient to compute the mass flows of the black-box models, as shown here. For the overall black-box model, we set a number of parameters: the excretion rates of solutes as per the median values in Table 2; the venous blood concentration of the plasma as per Table 1; and the flow rate of the arterial blood as 900 l/day [23, 34]. In addition, we chose the *amount* of water excreted to be the variable parameter, resulting in the *concentration* of solutes in the urine being variable. Once these variables have been allocated values, the flow rates of the various substances in the blood leaving the kidney can be determined as the difference between the arterial blood flow rate and the flow rate of urine.

A complete model of the flow rates in the two sub-processes, represented by two black-box models inside of the kidney, requires two more quantities. Firstly, we assumed the concentrations of the filtrate and efferent blood plasma are equal to that of the arterial blood, with the exception of the efferent plasma containing all the proteins and the filtrate containing none. Secondly, the mass flow rates are set by the filtration fraction of 20%, as discussed above. This completes the mass flow data for all the streams considered in this study, from which we derived the concentrations, volumes and mole fractions, as required for the thermodynamic calculations.

#### Gibbs energy flow calculations

Once the mass flow in the system can be described in terms of molecular flow rates and mole fractions, we can calculate the Gibbs energy (G). By definition, G includes all energy available for useful work and can include work done by pressure gradients, concentration gradients and chemical reactions [28]. At this stage, we exclude chemical reactions, and by extension exclude the metabolic energy consumed in the kidney from the model. This term can be added to the model if required, although with the data available at this stage, the metabolic energy can only be estimated to an order of magnitude and the sensitivities to variations in urine concentrations are unclear.

G can be calculated as [28]:

1 $$ G={\displaystyle \sum_{j=1}^n}{\mu}_j{N}_j $$

Where:

- *G* = Gibbs energy flow (W);
- *μ*_*j*_ = chemical potential (also known as partial molar free energy) of component *j* (J/mol); and
- *N* = molar flow rate of component *j* (mol/s).

In addition, the extent by which pressure affects chemical potential can be quantified as follows [28]:

$$ {\mu}_j\left({T}_1,{P}_2,{x}_j\right)={\mu}_j\left({T}_1,{P}_1,{x}_j\right)+{\displaystyle {\int}_{P_1}^{P_2}\overline{V_J}} d P $$

By assuming the liquids are incompressible, the equation becomes:

$$ {\mu}_j\left({T}_1,{P}_2,{x}_j\right)={\mu}_j\left({T}_1,{P}_1,{x}_j\right)+\overline{V_j}\varDelta P $$

Where:

- *P*_1_ = reference pressure, chosen as ambient pressure (Pa);
- *P*_2_ = system pressure (Pa);
- *T*_1_ = reference temperature, chosen as the body’s temperature, 310 K; and
- $\overline{V_J}$ = partial-molar volume of component *j* (m^3^/mol).

Furthermore, the effect of mixing in a mixture can be expressed as [28]:

$$ {\mu}_j\left({T}_1,{P}_1,{x}_j\right)={\mu}_j^0\left({T}_1,{P}_1\right)+ R T \ln \left({\gamma}_j{x}_j\right) $$

Where:

- *x*_*j*_ = molar fraction of component *j* in the total mixture (dimensionless);
- *μ*_*j*_^0^ = chemical potential of a pure component (J/mol);
- *γ*_*j*_ = activity coefficient of component *j* (dimensionless); and
- *R* = the ideal gas constant (8.3144621 J/mol.K).

These calculations were simplified by taking activity coefficients as unity, based on a review of available data [18, 24, 37]. Better approximations for the activity coefficients are required to obtain results that are more accurate. In addition, partial-molar volume $\left(\overline{V_j}\right)$ was approximated as pure component volumes (*V*_*j*_).

Putting the above equations together, we obtain:

$$ G={\displaystyle \sum_{j=1}^n}{N}_j\left({\mu}_j^0\left({T}_1,{P}_1\right)+{V}_j\varDelta P+ R{T}_1 \ln {x}_j\right) $$

Next we set a reference point at which pure chemical compounds have a standard chemical potential of zero, at a temperature of 310 K and a pressure of 1 bar. Assuming that there is no reaction inside the fluids, the system temperature is 310 K and all pressure measurements are in gauge pressure, such that *P*_2_ *-P*_1_ = *P*_*g*_, we obtain Eq. 2, below.

2 $$ G={\displaystyle \sum_{j=1}^n}{N}_j{V}_j{P}_{\mathit{\mathsf{g}}}+ R T{\displaystyle \sum_{j=1}^n}{N}_j \ln {x}_j $$

The first term on the right hand side of Eq. 2 is the Gibbs energy associated with the pressure of the liquid flowing through the kidney while the second term on the right hand side of Eq. 2 is the Gibbs energy associated with concentration, which is associated with mixing and separation. In the context of this study, Eq. 2 will be applied to study the energy differences between the streams entering and leaving the black-box models.

$$ \varDelta {G}_{total}={G}_{leaving}-{G}_{entering} $$

Here, the total change in Gibbs energy (∆*G*_*total*_) is equal to the combined work associated with changes in pressure and changes in the extent of separation:

$$ \varDelta {G}_{total}={W}_{pres}+{W}_{sep} $$

Where:

3 $$ {W}_{pres}={\displaystyle \sum_{j=1}^n}{N}_j{V}_j\varDelta {P}_{gj} $$

4 $$ {W}_{sep}= R T\ {\displaystyle \sum_{j=1}^n}{N}_j \ln \varDelta {x}_j $$

By using the calculated mass flow parameters and the available pressure data in these equations, we calculated the G associated with each stream. This served as a basis for analysing the total change in G (∆*G*_*total*_), the work associated with pressure change (*W*_*pres*_) and the work associated with separation (*W*_*sep*_). A sample calculation is available through the link in at the end of the paper.

#### Finding thermodynamic limits and optima

The G equation, Eq. 2, can be used to examine the work associated with points within the empirically measured stream property ranges from the input data. Firstly, in order for the process to be thermodynamically feasible, there must be available capacity for work. This implies the energy level associated with the streams leaving the system must be lower than that entering the system, which means ΔG_total_ < 0. We refer to the thermodynamic limit, accordingly, as ΔG_total_ = 0, which is the point where there is just sufficient G flowing into the system to produce the specified outlet concentrations.

We can furthermore determine the thermodynamic optimum by finding where the work requirement is minimised. The optimum is regarded as the point where ΔG_total_ is the most negative and excess or lost work is maximised.

#### Sensitivity analysis

In order for this study to present conclusive results and for future studies to build on it, it must consider a vast range of scenarios. Hence the sensitivity analysis calculates the dependency of separation work requirements on the excretion rate of the major substances in urine, namely sodium, potassium, urea and water. The sensitivity of the model under various conditions may be examined further by downloading the model using the link in at the end of the paper.

## Results

### Results obtained from the overall black-box model

**Fig. 2:**
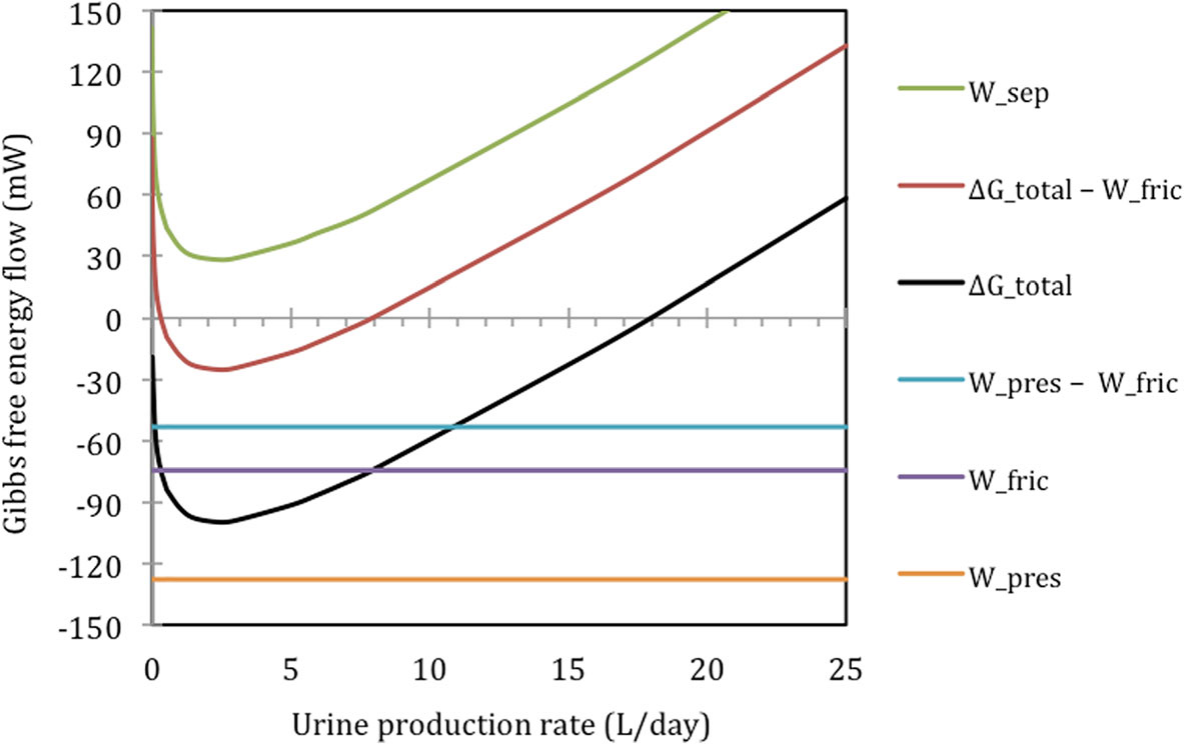
The Gibbs energy flow in or out of the process streams for different urine volume production rates, at a constant solute excretion of 731 mmol/day, as in Table 2. Variables depicted on the graph include total change in Gibbs energy (ΔG_total_), pressure work (W_pres_), separation work (W_sep_) and friction losses (W_fric_); these variables are described in Eqs. 2, 3, 4 and 5 respectively. Negative values for ΔG_total_ indicate that there is excess or lost work, which implies the stream parameters are achievable without additional work applied to the system

The first analysis of the overall black-box model shown in Fig. 1, involved varying the *amount of water* excreted while keeping the amounts of solutes excreted constant, such that the urine concentrations are effectively varied. We chose to report the G flow in mW, which can be converted to units of kJ/day if need be. Fig. 2 depicts the corresponding change in ΔG_total_. The daily urine volume ranges on the graph where ΔG_total_ < 0 are ranges where the work supplied by the pressure drop across the system is more than the work of separation.

On the above graph, the line labelled ΔG_total_ indicates that for the overall black-box model the total change in G is negative for urine volumes ranging from less than 1 ml/day up to 18 l/day. Over this range the pressure supplied to the kidneys by the heart theoretically supplies sufficient work for the urine production process to be spontaneous. If the energy from chemical reactions, associated with metabolic processes in the kidneys were added into the model, the total overall change in G would have been significantly more negative.

The line marked W_sep_ on Fig. 2 shows the work required to achieve the relevant urine concentrations used as the model input parameters. This work requirement is equal to the minimum energy required by a mechanism that operates at 100% efficiency. Less efficient mechanisms may be present in the human kidneys and thus the kidney may require more work in order to produce urine.

Even though the results indicate that the potential work that can be done from the total change in G (∆G_total_) is sufficient to produce a range of urine volumes up to 18 l/day, there may be additional energy costs for the real system. For example, it is known that there are pressure drops due to friction in the afferent and efferent blood streams, before and after the glomeruli, as discussed in the pressure data section. In these sections (marked as “valves” in Fig. 1), no separation work is done and the energy is lost to friction (an irreversible processes), which implies it cannot be used to power separation later. Consequently, additional results are included on Fig. 2, using the variables below, to differentiate between pressure work that can be applied to drive separation and that which is lost to friction:

5 $$ \begin{array}{c}\hfill {W}_{pres}={W}_{pres,\kern0.75em  afferent}+{W}_{pres,\kern0.75em  glom}+{W}_{pres,\kern0.75em  efferent} + {W}_{pres,\kern0.75em  tubules}\hfill \\ {}\hfill {W}_{fric}\kern0.75em  = {W}_{pres,\kern0.75em  afferent}+{W}_{pres,\kern0.75em  efferent}\hfill \end{array} $$

When these known pressure losses are taken into account, the lines ∆G_total_ and W_pres,_ are displaced upwards by 74 mW, while the work requirement to change concentrations, W_sep_ remains unaffected. With this energy loss accounted for, the range of flow rates of urine where ∆G_total,_ <0 decreases to between 0.31 l/day and 8.1 l/day, as shown in Fig. 2. However, regardless of the pressure lost to friction or other inefficiencies, the work requirement for urine production will be a *minimum* at a urine production rate of 2.39 l/day for a solute excretion rate of 0.731 mol/day, as shown in Fig. 2.

### Results for the two-compartment black-box model

With the two-compartment black-box model, it is possible to differentiate between the work of glomerular filtration and the work of filtrate recovery. Again, these calculations show the required or the available work rate, calculated as the change in the flow in G across the glomerular compartment and the tubular system, namely ∆G_glom_ and ∆G_tub_ respectively.

**Fig. 3:**
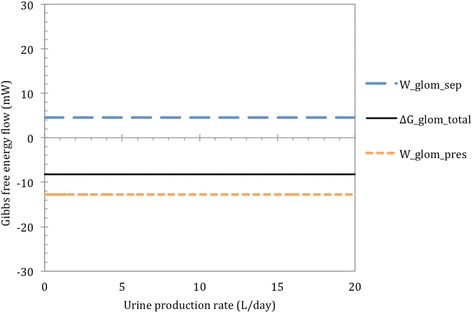
The Gibbs energy flow in or out of the process streams during glomerular filtration for different urine volume production rates, at a constant solute excretion of 731 mmol/day, as in Table 2. The blue line is the separation work across the glomeruli; the orange line is the pressure work and the black line is the sum of these two. Negative values for G indicate excess potential to do work

The calculation results for the glomeruli are given in Fig. 3 and we again considered both the pressure and mixing terms as well as the total for each box. We made the assumption of a constant 20% filtration fraction in the glomerulus, which is the reason any change in urine production volume does not affect the G terms in the glomeruli. We find that W_glom,sep_ = 5 mW while W_glom,pres_ = -13 mW meaning that ∆G_glom_ = -8 mW. Thus, the model is in accord with conventional understanding of how the glomeruli work: sufficient power is available from the pressure drop across the glomeruli to produce the filtrate.

**Fig. 4:**
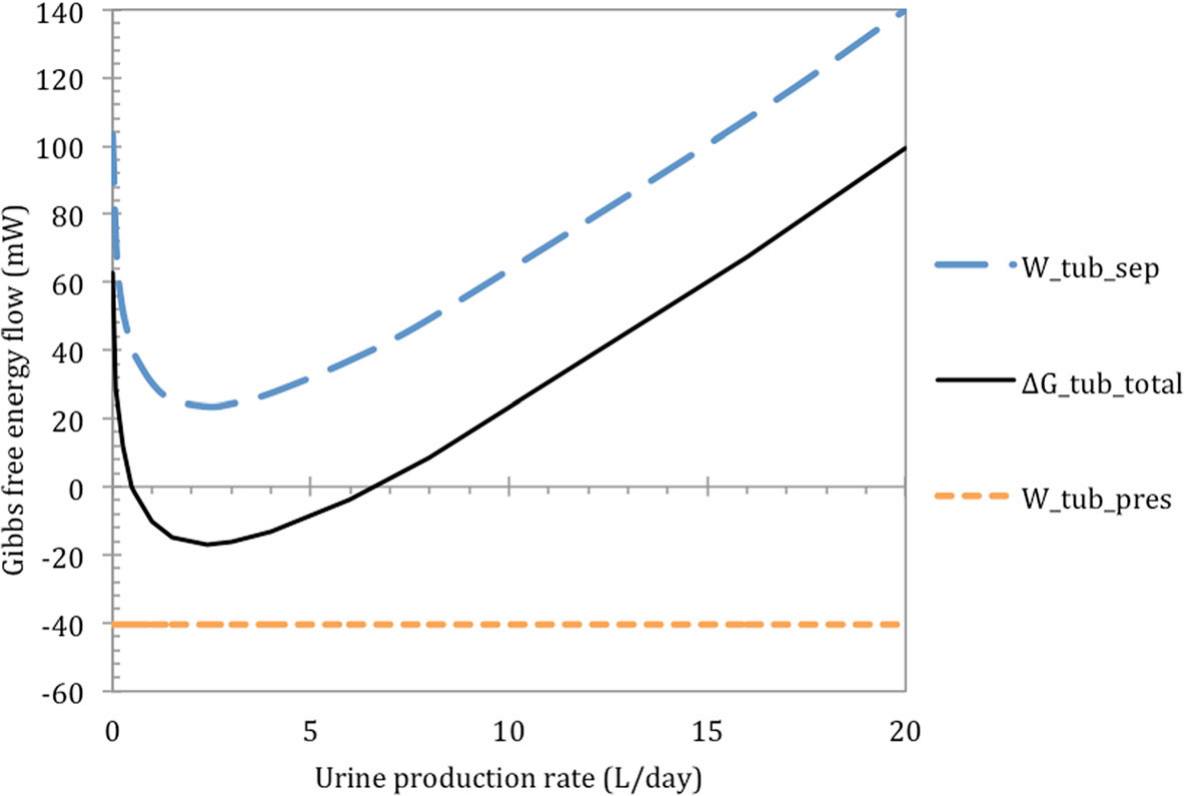
The Gibbs energy flow in or out of the process streams during tubular reabsorption and its components for different urine volume production rates, at a constant solute excretion of 731 mmol/day, as in Table 2. As in the previous graph, the blue curve corresponds to separation work, the orange line to pressure work and the black curve to the total. Negative values for G indicate excess potential to do work

In the tubular system, W_sep_ does depend on the urine production rate, as can be deduced from the process schematic in Fig. 1 and can be seen in the results shown in Fig. 4. W_sep_ reaches a minimum at 23 mW, when 2.39 l/day of urine is produced. Because W_pres_ for the tubular system is constantly at -41 mW, ∆G_tub, total_ is -17 mW at the minimum. In this theoretical system, there is excess capacity for work when ∆G_tub, total_ is negative, when urine production is between 0.5 l/day to 6.5 l/day. Despite availability of this energy, the complexity of renal tubules requires the consideration of multiple other factors to determine to what extent, if at all, this energy from pressure is available for further urine formation. This is explored further in the discussion.

### Sensitivity analysis: The effect of varying solute excretion

The overall black-box model is dependent on more factors than those considered in Figs. 2, 3 and 4 above, such as variations in the excretion rate of specific solutes or the concentration of the blood plasma fed into the system. In this sub-section, we first consider the effect of varying solute excretion, followed in the next sub-section by the variation of plasma concentration. For these results, we analysed separation work (W_sep_) in the overall system and its dependency on the rate at which different substances are excreted. The results are depicted as contours of W_sep_ in a two-dimensional plain. These contours in the graphs are the boundaries of the attainable region for different amounts of energy applied. For example, W_sep_ for the overall system is less than 45 mW within the “+45 mW” contour. We have not included W_pres_ in the contours such that we can have clarity that the quantity of work required to separate the substances is independent of which mechanism carries out the work.

**Fig. 5:**
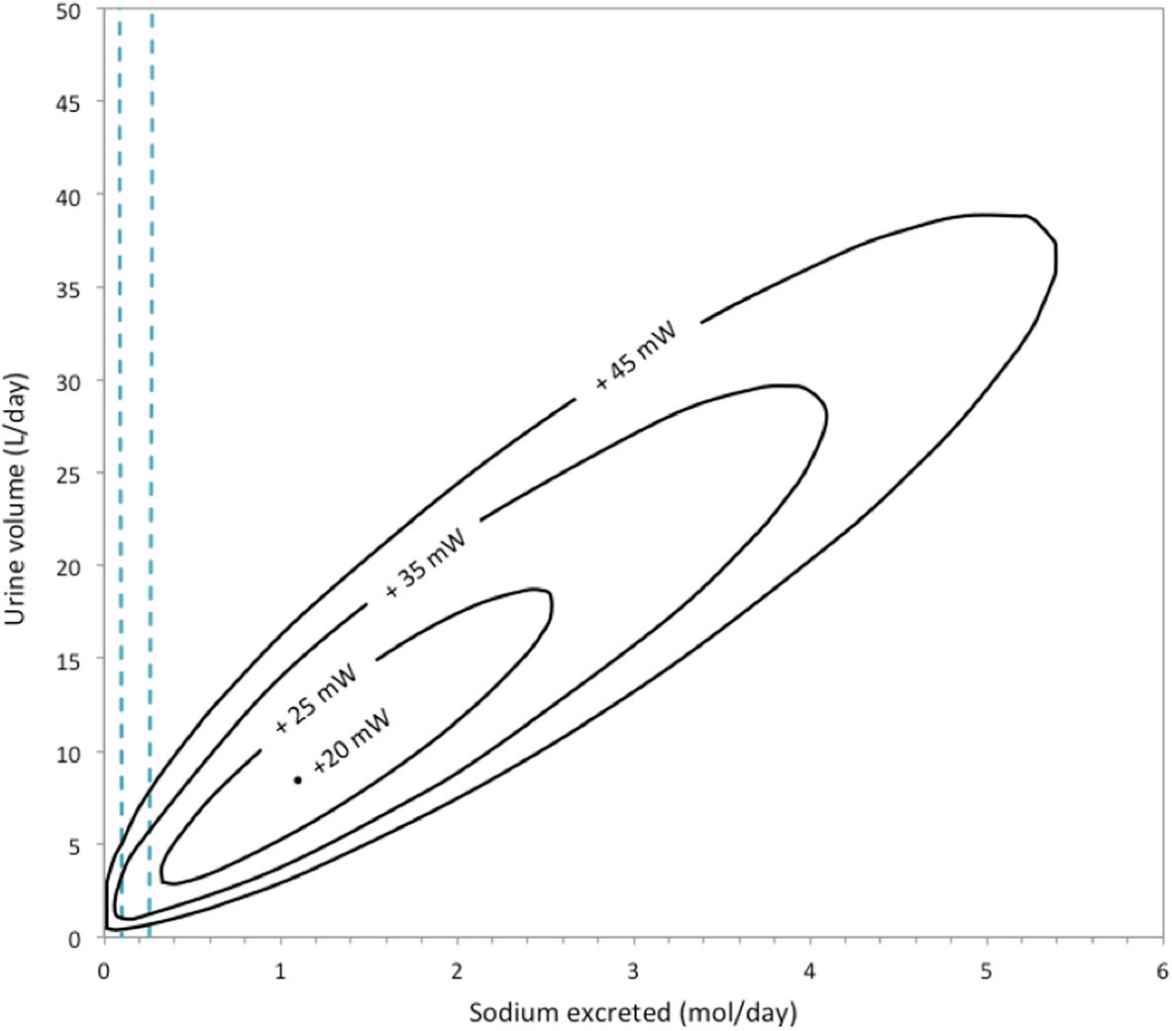
A Gibbs energy contour map, depicting the variation of separation work (W_sep_) in the overall kidney, as sodium chloride and water excretion is varied, with the dashed lines indicating the typical reference range of excretion rates in humans. The concentrations of all other solutes and the reference range concentration are as in Table 2

**Fig. 6:**
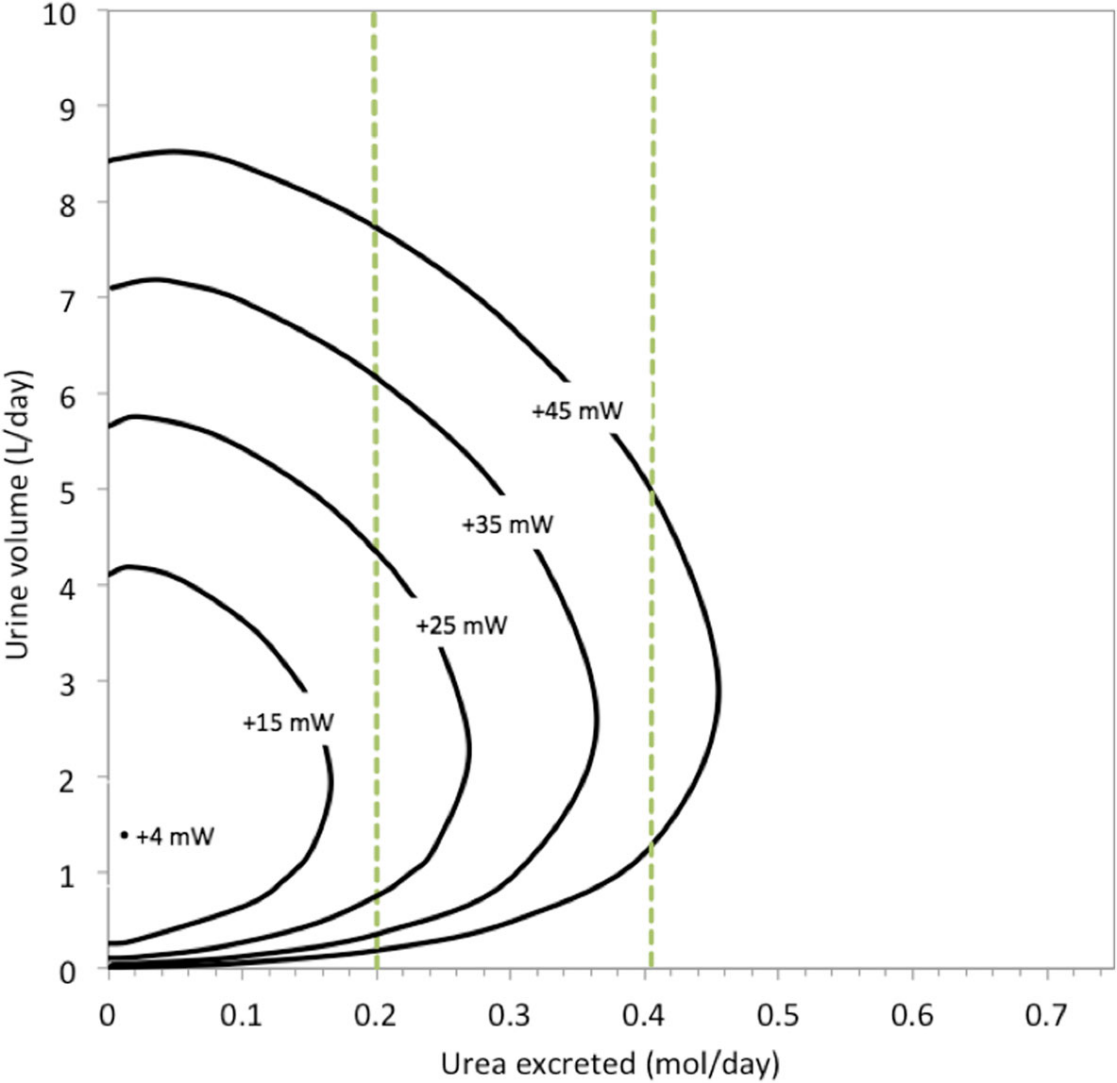
A Gibbs energy contour map, depicting the effect of varying the amount of water and urea excreted on separation work (W_sep_) in the overall kidney, with the dashed lines indicating the typical reference range of excretion rates in humans. The concentrations of all other solutes and the reference range concentration are as in Table 2

**Fig. 7:**
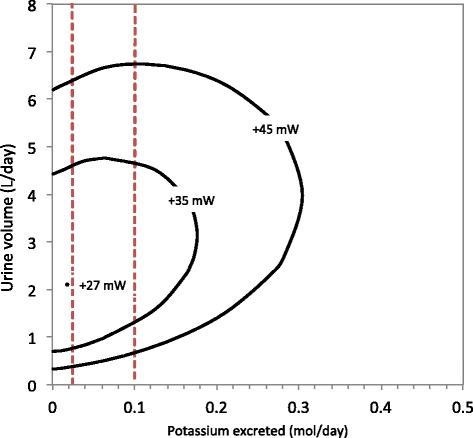
A Gibbs energy contour map, depicting the effect of varying the amount of water and potassium chloride excreted on separation work (W_sep_) in the overall system, with the dashed lines indicating the typical reference range of excretion rates in humans. The concentrations of all other solutes and the reference range concentration are as in Table 2

Figure 5 depicts the effect of varying the amount of sodium and chloride ions excreted on the urine volume produced, while urea excretion is varied in Fig. 6 and potassium chloride excretion is varied in Fig. 7. The figures also include reference ranges for typical human excretion rates, as given in Table 2, which serve to place the results into perspective.

In the above graphs, the water excretion rate associated with minimum work shifts dramatically: from 8.4 l/day in Fig. 5 to 1.4 l/day in Fig. 6 to 2.1 l/day in Fig. 7. These volumes are the optima predicted by the model and whether the kidneys do function accordingly remains to be established by clinical research. We further observe that within the ranges of normal human excretion, and within the reference ranges, the optimum lies between 2 and 3 l/day in all of the above graphs.

The optimum point is lowest on Fig. 6. At this point, the work requirement is only 4 mW, which indicates there would be no need to do work on the system to produce the specified urine concentrations. Here the urea concentration in the urine is close to that of the blood: in the urine, urea concentration is 4.2 mmol/l, while it is 4 mmol/l in the blood.

Beyond the results depicted graphically, there are additional numerical results that are of interest. The calculations depicted in Fig. 5 also show that if 45 mW were applied doing separation work, it is theoretically possible to excrete approximately 21% of the water filtered out of the blood by the glomeruli: that is 37.8 l/day of 180 l/day. In contrast, the same quantitative amount of work is sufficient to excrete 62% of the filtered amount for urea. For potassium and sodium ions the maximum excretion rates are about 40% and 43% of the amount in the original filtrate, respectively.

At one point on the +45 mW contour, the concentration of sodium is 2.8 times higher in the urine than in the blood. What is more, elsewhere on the same +45 mW contour the sodium concentration in the urine goes down to one twentieth of the concentration in the blood. For urea this ratio of concentrations ranges from 432 to 0.03 times that in blood. This means that the boundary lines for the thermodynamically attainable region in the graphs are not always determined by the concentration gradient between the blood and the urine. At some point other substances’ concentration differences between the blood and filtrate would limit urine production.

### Sensitivity analysis: Varying blood plasma concentration

The model offers an opportunity to build on the work of Newburgh [22], who first indicated a possible thermodynamic feedback that changes the work requirements for urine excretion, as the blood concentration is affected by the excretion of urine. To explore this impact, we carried out another analysis to determine the effect of plasma dilution on urine production work requirements.

**Fig. 8:**
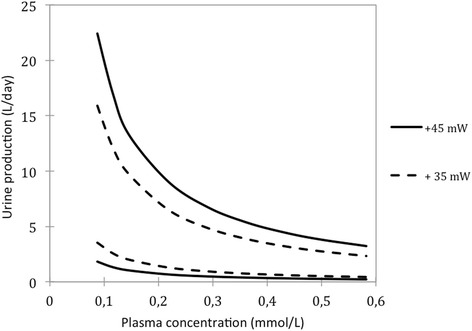
The required amount of separation work (W_sep_) required in the overall kidneys to produce a volume of urine, relative of plasma solute concentrations for the blood entering the kidneys

**Fig. 9:**
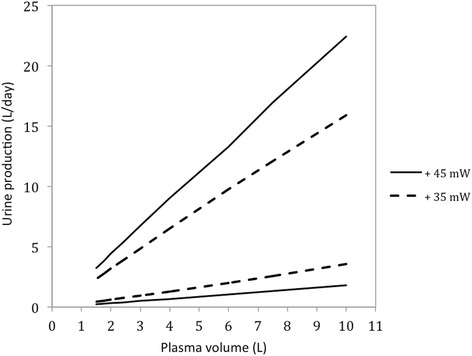
The separation work requirement (W_sep_) for different urine production volumes in the human kidneys, when a person’s blood plasma volume is varied while the amount of blood solutes is kept constant. In this graph, a person who would otherwise contain 3 litres of blood plasma and 291 mmol/l of solutes, as in Table 1, has his blood diluted or concentrated through the addition or removal of water. In other words, this graph effectively depicts changes in the blood plasma concentration fed into the system versus the work requirement associated with urine production volumes

Figures 8 and 9 show the results obtained from two different perspectives. In Fig. 8, the plasma solute concentrations were varied by the addition or removal of water. In order to arrive at Fig. 9, we first assumed that the standard plasma volume is 3 litres [9], and then we calculated the standard amount of solutes in the body. This can be done from the concentrations listed in Table 1. Finally, we varied the plasma volume while keeping the blood solutes in the body constant, to construct the work requirement contour lines on the graph.

The results depicted in Fig. 9 show that if the plasma volume increases by 100 ml from 3 litres to 3.1 litres, then the maximum feasible urine volume increases by 190 ml, if 45 mW is applied constantly doing separation work. Alternatively, if the plasma volume were decreased from 3 litres by 100 ml, then the maximum feasible urine volume producible with 45 mW of work would decrease by 190 ml. With 35 mW applied, the maximum feasible urine volume changes only by 120 ml per 100 ml extra plasma.

## Discussion

### The overall model

The model correlates well with other studies. According to Fig. 2, where the solute excretion rate is 731 mmol/day, the work requirements are minimum at a urine production rate of 2.39 l/day, at a chemical work requirement of 28 mW. This corresponds to the optima calculated by Newburgh [22]: the optimum urine production rate was calculated as 2.36 l/day and the chemical work requirement as 38 mW, at a solute excretion rate of 780 mmol/day (which comprised of 400 mmol/day urea and 190 mmol/day of both sodium and chloride ions).

These theoretical calculation results from simplified models of the kidney do correspond to empirically measured results. Indeed, the urine volume that minimises work requirements is approximately equal to the water intake requirement for a person. A review of empirical studies shows that a water intake of 2.5 l/day is needed by the average inactive man to maintain constant levels of hydration over several days [30]. This study also indicates water intake requirement can increase to 3.7 l/day for men who are more physically active, which may be attributed to increased perspiration [30].

Actual energy consumption in the kidneys is two orders of magnitude larger than the theoretical amount of energy needed to do the work associated with separating a urine stream from blood. The kidneys use about 7% of the oxygen consumed by the body [32], and the average basal metabolic rate for a person is about 80 W [9]. Hence, the average metabolic rate of the kidney can be estimated as 6 W.^1^ At least 60% of this amount is thought to be used to drive mass transfer for urine production [32]. In contrast, as shown in Fig. 2 and as corroborated in the sensitivity analysis results, the effective amount of work done through changing concentrations, that is in separating the streams, is in the order of 54 mW or less. Moreover, the calculations from Fig. 2 show that the heart supplies 128 mW to the kidney via blood pressure.

The results obtained from the first overall model of the kidneys, Fig. 2, show that there is sufficient capacity for work to produce urine – without any additional energy from metabolism or chemical reactions. The total G change over the entire process is quantitatively sufficient for urine production to be spontaneous for volumetric rates between 0.31 l/day and 8.1 l/day. Thus, there is a possibility that some real system could perform the same work as the kidneys, with the same feed and exit streams, without needing any of the 6 W of energy available from metabolic processes. It is not known from these calculations, however, what mechanisms would make up a system that utilises available pressure work (W_pres_) to drive separation (W_sep_).

### The two-compartment model

By splitting the model into two compartments, as in Fig. 1, the model becomes more representative of the human kidneys: it simulates the creation of a protein-free filtrate, just like the kidneys, and it accounts for the pressure lost in order to control the afferent and efferent blood pressures. With this model we can differentiate between the work done in the glomeruli and the work done while recovering the filtrate in the tubular system. More importantly, we can determine where energy is lost and where it is available.

Filtration takes place in the first compartment, which represent the glomeruli. Figure 3 shows that separation work takes place at a rate of +5 mW, while work is being done through pressure drop at a rate of -13 mW. This implies sufficient G for a standard filtration process, wherein a spontaneous decrease in pressure drives the non-spontaneous process of creating a concentrate.

Now that the concentration gradient created by glomerular filtration has been quantified as a 5 mW work investment, we know the maximum quantity of work that can be done with the concentration gradient is 5 mW. The concentration gradient between the efferent blood and the filtrate could thus possibly be used to drive the recovery of filtrate later in the system, as discussed by Letts et al. [16].

The work requirements for the second compartment, the tubular system, reaches a minimum point at 2.39 l/day similar to the results of the overall system, as shown in Fig. 4 and Fig. 2 respectively. Since the separation work goes down to +23 mW, while the pressure work is constant at -41 mW, it seems there is sufficient change in G over the tubular system for it to concentrate the filtrate without the application of additional work. However, there is no obvious mechanism to utilise the pressure drop for driving mass transfer, given that the pressure gradient at the start of the tubular black box is such that flow would be driven from the efferent blood to the filtrate.

### Solute excretion limits

The sensitivity analysis of the solutes excreted further showed the incongruity between the 6 W of metabolic energy consumption of the kidneys and the amount of separation work done. Separation work done in the overall process only amounts to 45 mW when extreme amounts of a particular solute are excreted. In addition, the results show that when sodium, potassium and urea excretion are at the maximum of their reference ranges, while urine volume is low at 1 l/day, the separation work requirement is only 54 mW. Other researchers have similarly concluded that the overall kidneys’ separation work is between 19 and 44 mW [1, 22]. With this study, we thus gain further confidence in the conclusion that the separation work requirement is relatively small.

By correlating extreme excretion rates, which have been measured empirically in medical cases, with specific model results, it is possible to further assess the maximum work capacity of the kidneys. There have been reports of urine production volumes of up to 20 l/day in humans [9] and the model’s results show at least two ways in which such a high urine production rate could be achieved. Figure 5 shows that an average human can produce 20 l/day of urine, with less than 35 mW allocated to the recovery of filtrate in the tubular system, if about 3 mol/day of sodium chloride were available for excretion. Alternatively, solute excretion rates could remain normal if the plasma concentration is diluted to at least 0.09 mol/l, as shown in Fig. 8, while 45 mW is applied in the tubular system. The latter case may relate to a clinical condition known as hypotonic hyponatremia, during which diluted plasma is associated with excessive urine production [12].

Other extreme cases are the known minimum water excretion rate and the maximum urine concentration. The lower daily urine volume limit is known as the “obligatory urine” production rate. It is roughly 0.5 l/day for the average adult [9]. Such a low volumetric flow rate can be achieved with less than 45 mW of separation work, when solute excretion for each substance is at the mean value of the reference ranges, such that the total solute excretion is 731 mmol/day, as in Fig. 2. Figures 5, 6 and 7 also show scenarios in which 0.5 l/day of urine is produce with 45 mW of separation work or less.

This amount of work is also sufficient to reach the maximum urine concentration achievable by humans, which is 1.2 mol/l [29] as mentioned earlier. As shown in Fig. 2, 45 mW of work is sufficient to produce a concentration of 1.462 mol/l, with the solute excretion set at 0.731 mol/day.

Note that it is possible to produce a smaller daily volume of urine, with the same rate of doing work, by reducing the amount of potassium chloride or urea excreted. Decreasing urea excretion to the bottom of the reference range will drop the minimum urine flow rate to 0.2 l/day, while implementing the same change on potassium chloride excretion brings the lower limit down to 0.4 l/day. This is clear from the 45 mW contours in Figs. 6 and 7.

Lastly, we will mention one more medical condition that can be used to quantify the exact limitation of the kidneys to do separation work. It can be seen in Figs. 5, 6 and 7 that the energy demands for solute excretion increase more for increased urea or potassium chloride excretion, than for sodium chloride excretion. Abnormally high excretion rates do occur during a pathological condition called rhabdomyolysis: muscle damage elevates the need for urea and potassium excretion. In extreme cases of rhabdomyolysis a patient can go into acute renal failure [11]. On the one hand, the removal of urea and potassium through hemodialysis or continuous hemofiltration has been shown to be an effective treatment for renal failure in such cases [11]. On the other hand, according to our model, in extreme cases of rhabdomyolysis there would be a greater than normal energy requirement. Thus, data collected from patients who suffer acute renal failure owing to rhabdomyolysis could provide an opportunity for future researchers to investigate the exact separation limitations of the kidneys.

### Feedback from plasma dilution

Newburgh [22] first suggested from his calculations that in a scenario where higher solute excretion rates are required, the accumulation of solutes in the body could reduce the energy requirements for renal excretion. This is in agreement with the results from this study. Figure 8, in particular, shows how the changes in blood concentration can affect the optimal and feasible urine volumes. This figure shows that decreases in blood concentration would cause increased urine volumes, while increases in blood concentration decrease the urine volume. The implication is this: if the kidney excretes too much water from the body and the blood plasma is consequently concentrated, then there is an automatic thermodynamic feedback that limits the urine volume. Conversely, if its ability to excrete is impaired, an accumulation of substances will reduce the energy requirement for excretion and thus facilitate excretion.

As a thought experiment that illustrates this point, one can imagine a perfectly hydrated person, with 3 litres of blood plasma, who rapidly drinks 1 litre of water. If one assumes that the total volume of water is absorbed into the blood, without entering other compartments of the body, the model shows that diluting the blood to this extent will increase the water excretion rate possible with 45 mW of separation work by 1.9 l/day. Hence, the system response to increased water ingestion is to excrete the extra volume ingested automatically, unless a smaller amount of energy is applied to do separation work. Figure 9 shows this relation is linear and decreases in blood concentration plasma similarly decrease urine volume.

There are many more factors to consider in a system as complex as human physiology. From a physiological perspective, for example, not all the additional water may be taken up in the blood. From a thermodynamic perspective, the extent of this feedback would be dependent on a person’s initial plasma volume. More measurements from empirical experiments are required for a conclusive analysis on the relation between water ingestion and renal workload. Nonetheless, as our first approximation has demonstrated, such thermodynamic feedback might be utilised by the body.

### Additional considerations

There are factors that will make the energy required in the kidney more than the effective amount of separation work done, but the possibility also exists that the work requirement in the tubular system may be less than estimated. Factors that could increase the energy requirement include inefficiencies such as the diffusion of ions back into the filtrate [5], as well as pressure losses due to friction. On the other hand, non-ideal behaviour of plasma proteins could potentially increase the energy storage in the glomeruli: a non-linear increase in the blood’s osmolality would increase the concentration gradient and decrease the energy requirement for filtrate recovery in the tubular system.

The model’s results show that in the glomeruli 13 mW of power is spent via pressure drop, while creation of a protein-free filtrate makes 5 mW available in the form of a concentration gradient. Thus, 38% of the pressure drop in the glomeruli is invested in the creation of a protein-free filtrate. However, the non-ideal behaviour of proteins, particularly serum albumin, has not been taken into account and it may significantly affect the efficiency of energy storage. Consider, for example, Cameron et al. [3] proposal that serum albumin’s dynamic behaviour could provide the driving force for the movement of water into and out of cells. While Weinstein [39] has studied the effect of this protein on renal processes, little attention has been given to its dynamic behaviour. There are more phenomena related to the functions of proteins in the kidney that have yet to be studied [7, 17]. Ultimately, all of these effects can be accounted for by the activity coefficient (*γ*) in the G, as shown in Equation 2. It is recommended that future models include this complexity.

## Conclusions

This study has produced results in agreement with previous theoretical and empirical studies, and then proceeded further to analyse a vast array of scenarios for urine concentrations. In particular, large amounts of data have been generated describing the variation of separation work requirements caused by changes in sodium, potassium and urea excretion. Although these numerical results may provide more insights when combined with additional empirical evidence, this paper builds arguments for two main conclusions: the first relates to the amount of energy required, while the second relates to the sensitivities of the system.

We conclude that the separation work requirement (around 20 to 54 mW) is two orders of magnitude smaller than the metabolic energy consumed by the kidneys (6 W), and less than half of the total work available from pressure (128 mW). The black-box models used in this study do not indicate how this energy might be utilised or whether the human kidneys could utilise this energy. However, it is theoretically possible for a system to exist that applies only the pressure from afferent blood to produce the same urine and purified blood that the kidneys produce.

An investigation into the model’s sensitivity to other parameters showed unexpected thermodynamic characteristics of the system. The results indicated that more dilute plasma fed into the kidney results in lower energy costs for larger urine volumes. This effect is such that for each additional 100 ml of water by which the plasma is diluted, the feasible urine volume increases by 190 ml, if the quantity of work associated with separation is kept constant at 45 mW. More data from empirical experiments are required to determine whether this renal control mechanism is actually used by human kidneys.

Further progress may be made with the line of investigation used in this study, through more detailed modelling, specifically by relating plasma concentration to urine production or incorporating the dynamic behaviour of plasma proteins. Indeed, we cannot claim to have a complete understanding of the glomerular energy storage mechanism, which entails the creation of a protein concentration gradient, without considering data on the non-ideal behaviour of human serum albumin.

## Appendix

### Summary of assumptions

List of assumptions:

- The model is based on the physical functions of a healthy human being.
- The components not included in the model do not significantly affect the thermodynamics of the system.
- There is no accumulation or depletion of substances inside the kidney, over the time period in consideration.
- Concentrations of sodium, potassium, calcium, chloride ions, bicarbonate ions, urea, creatinine and glucose are the same in the glomerular filtrate and the efferent blood, immediately after the glomeruli.
- The pressure losses due to friction are constant.
- The energy calculations have a reference point of compounds at 310 K.
- The pressure values are gauge pressure, and the ambient pressure is 1 bar.
- The urine pressure is equal to that of the renal veins.
- There is no chemical reaction in the system.
- All mixtures are ideal, with no excess Gibbs energy.
- The activity coefficients of all substances are unity.
- The change in solution density due to ionic interactions is negligible.
- The concentration of water for urine is 55.14 mol/l, as it would be for pure water at 310 K [27], but if there are proteins in the solution, they will dilute the water.
- Fluids in the system are incompressible.

### Protein data

**Table 4:** Protein property data used in this study

Protein	Property	Value
Human serum albumin	Molecular weight	66 248.3 g/mol (Peters, 1975) [25]
	Density	1.36 g/ml (Peters, 1975) [25]
	Serum level	42 ± 3.5 g/l (Peters, 1975) [25]
Fibrinogen	Molecular weight	340 000 g/mol (Doolittle, 1975) [6]
	Density	1.4 g/ml (Doolittle, 1975) [6]
	Serum level	3 ± 1 g/l (Doolittle, 1975) [6]
Immunoglobulin	Average molecular weight	212 000 g/mol (Putnam, 1977) [26]
	Density	1.4 g/ml [Assumed]
	Serum level	16 ± 4 g/l (Putnam, 1977) [26]

## Abbreviations

∆G: Change in Gibbs energy
G: Gibbs energy
_glom_: in the glomeruli
_pres_: associated with pressure
_sep_: associated with change in concentrations, i.e. separation.
_tub_: in the tubular system

## Footnotes

1 The energy consumption of the kidneys is measured as 21.3 W/kg [8, 36]. When this figure is multiplied by the weight of two average kidneys, which is 0.3 kg combined [9], the total energy consumption is 6 W.
